# Inflammatory and cardiometabolic markers at presentation with first episode psychosis and long-term clinical outcomes: A longitudinal study using electronic health records

**DOI:** 10.1016/j.bbi.2020.09.011

**Published:** 2021-01

**Authors:** Emanuele F. Osimo, Benjamin I. Perry, Rudolf N. Cardinal, Mary-Ellen Lynall, Jonathan Lewis, Arti Kudchadkar, Graham K. Murray, Jesus Perez, Peter B. Jones, Golam M. Khandaker

**Affiliations:** a Department of Psychiatry, University of Cambridge, Cambridge, UK; b Cambridgeshire and Peterborough NHS Foundation Trust, Cambridge, UK; c MRC London Institute of Medical Sciences, Institute of Clinical Sciences, Imperial College, Hammersmith Campus, London, UK; d Norwich Medical School, University of East Anglia. Norwich, UK; e Applied Research Collaboration East of England, National Institute for Health Research (NIHR), UK

**Keywords:** Psychosis, First episode psychosis, Inflammation, Metabolism, Basophils, Triglycerides, Clinical outcome, Early intervention, Longitudinal, Schizophrenia

## Abstract

Approximately one third of patients presenting with a first episode of psychosis need long-term support, but there is a limited understanding of the sociodemographic or biological factors that predict this outcome. We used electronic health records from a naturalistic cohort of consecutive patients referred to an early intervention in psychosis service to address this question. We extracted data on demographic (age, sex, ethnicity and marital status), immune (differential cell count measures and C-reactive protein (CRP)) and metabolic (cholesterol, triglycerides, glucose, glycated haemoglobin, blood pressure, body mass index (BMI)) factors at baseline, and subsequent need for long-term secondary (specialist) psychiatric care. Of 749 patients with outcome data available, 447 (60%) had a good outcome and were discharged to primary care, while 302 (40%) required follow-up by secondary mental health services indicating a worse outcome. The need for ongoing secondary mental healthcare was associated with high triglyceride levels (adjusted odds ratio/OR = 7.32, 95% CI 2.26–28.06), a low basophil:lymphocyte ratio (adjusted OR = 0.14, 95% CI 0.02–0.58), and a high monocyte count (adjusted OR = 2.78, 95% CI 1.02–8.06) at baseline. The associations for baseline basophil (unadjusted OR = 0.27 per SD, 95% CI 0.10–0.62) and platelet counts (unadjusted OR = 2.88, 95% CI 1.29–6.63) attenuated following adjustment for BMI. Baseline CRP levels or BMI were not associated with long-term psychiatric outcomes. In conclusion, we provide evidence that triglyceride levels and several blood cell counts measured at presentation may be clinically useful markers of long-term prognosis for first episode psychosis in clinical settings. These findings will require replication.

## Highlights

• ~60% of patients with a first episode of psychosis have a favourable psychiatric outcome.
• Demographics or psychiatric diagnoses do not differ between patients by outcome.
• Baseline triglyceride levels were directly associated with an unfavourable outcome.
• Baseline monocyte, lymphocyte and platelet counts were directly associated with an unfavourable outcome.
• Baseline basophils and basophil:lymphocyte ratios were directly associated with a favourable outcome.
• Baseline CRP levels and BMI were not associated with long-term psychiatric outcome.

## 1 Introduction

Schizophrenia is a serious mental illness affecting approximately 40 million people globally (Saha et al., 2005). Early intervention in psychosis currently represents the gold standard of care for people presenting with a first episode of psychosis (FEP) (McGorry, 2015). In this model, a specialised multi-disciplinary team delivers pharmacological and psychological interventions, family and social support, support with employment, and physical health care checks for up to 5 years to patients diagnosed with a FEP, thus reducing the duration of untreated psychosis (Marshall et al., 2005) and improving long-term psychiatric outcomes (Penttilä et al., 2014, Perkins et al., 2005). FEP patients receiving care from EI services (EIS) have a generally positive outlook. Previous evidence suggests that 42% will have a “good outcome”, such as remission, and 27% will have “poor outcomes”, such as needing further secondary (specialist) care input (Menezes et al., 2006). Furthermore, 13.5–19.2% show the best possible outcome, i.e. full recovery (Jääskeläinen et al., 2013, Wunderink et al., 2009).

It is currently difficult to predict the prognosis of an individual patient with FEP at the time of presentation to an EIS. Existing studies have reported certain baseline factors to be associated with subsequent poor clinical course/outcome, such as long duration of untreated psychosis (Penttilä et al., 2014, Perkins et al., 2005), poor pre-morbid functioning (White et al., 2009), prevailing baseline negative symptoms (White et al., 2009), lower IQ (Leeson et al., 2009) and persistent substance abuse (Barnett et al., 2007).

Biomarkers could aid better prediction of treatment outcomes in patients with FEP. There is cross-sectional evidence that drug-naïve FEP patients show evidence of inflammation, including elevated C-reactive protein (CRP) and inflammatory cytokine levels (Pillinger et al., 2019), elevated neutrophil and monocyte counts (Jackson and Miller, 2019), and elevated neutrophil to lymphocyte ratios, or NLR (Moody and Miller, 2018). Similarly, meta-analytic evidence suggests metabolic alterations in FEP, such as impaired glucose tolerance, insulin resistance (Perry et al., 2016), and high triglycerides (Pillinger et al., 2017). Studies showing a longitudinal association between childhood inflammation and future risk of psychosis in adulthood provide evidence that raised inflammatory markers in patients with psychosis are not solely a consequence of their illness (Khandaker et al., 2014, Metcalf et al., 2017, Wium-Andersen et al., 2014). Furthermore, Mendelian randomisation studies also support a potentially causal association between lymphocyte counts, IL-6 and CRP levels and psychosis (Astle et al., 2016, Hartwig et al., 2017, Khandaker et al., 2017).

Recently, Nettis and colleagues have reported that a combination of inflammatory (high CRP) and cardiometabolic markers (elevated triglycerides and body mass index (BMI)) at baseline could help predict clinical outcomes at follow-up (at about one year) in a small sample of 42 FEP patients (Nettis et al., 2019). This study aims to extend these potentially clinically useful findings by including a larger sample of patients from an EIS, a larger and more detailed set of predictor variables, and by extending the follow-up.

In this study, we examined longitudinal associations between immune and cardio-metabolic markers at baseline and clinical outcomes at 1–5 years in FEP patients using real-world clinical data from electronic health records (EHR) from an EIS in England. We also explored potential correlations between cardiometabolic and immune markers in FEP.

## 2 Methods

### 2.1 Setting and sample selection

CAMEO is a specialist EIS for people with FEP living in Cambridgeshire, Fenland and Peterborough, geographically defined areas in East of England. Referrals are accepted from multiple sources including general practitioners (GPs), mental health services, school and college counsellors, relatives, and self-referrals. People referred to CAMEO receive a comprehensive clinical assessment and are offered a physical examination and venepuncture for inflammatory and cardiometabolic markers. This study includes all patients who have been accepted by CAMEO between January 2013 and November 2019. The CAMEO service accepts patients who present with a psychotic episode for the first time, or if previous psychosis was either untreated or treated with antipsychotic medication for <6 months. It accepts people presenting with psychotic symptoms from any cause, including drug-induced psychoses and affective psychoses (including ICD-10 codes F06.0-2, F20-F31, F32.3, F33.3, F53.1). The EIS patients’ age range was 14–35 years until 1 April 2016, then became 14–65 years. All FEP patients were followed up for up to 3 years. We excluded patients who had not completed the EIS intervention or had moved out of area, as these groups did not have outcome variables available.

For this study, FEP patients were identified by carrying out an anonymised search of EHRs held by Cambridgeshire and Peterborough NHS Foundation Trust (CPFT), the UK National Health Service (NHS) provider of mental health services to the region. These patients received EI from CAMEO between 2013 and November 2019 (inclusive). Patient records were de-identified electronically using the Clinical Records Anonymisation and Text Extraction (CRATE) tool purpose built for research based on EHR data (Cardinal, 2017), and transferred into a research database with NHS and institutional approvals (UK NHS National Research Ethics Service references 12/EE/0407 and 17/EE/0442).

### 2.2 Extraction and coding of categorical information

#### 2.2.1 Sociodemographic information

For each patient, we extracted the following categorical information directly from the database: approximate date of birth/ age in years, sex, ethnicity, marital status, EIS involvement start and end dates.

#### 2.2.2 Primary outcome (psychiatric illness course)

Patients were categorised into two groups reflecting their psychiatric illness course, based on their clinical destination after discharge from the EIS:

• Discharged to primary care (GP) with no onward referrals to community mental health teams upon discharge, or within 2 years of discharge (so-called good outcome/ illness course).
• Continued involvement of secondary mental health services including one or more referrals to community mental health teams, crisis team or hospitalization within 2 years of discharge (so-called poor outcome/ illness course).

#### 2.2.3 Exploratory outcomes (ICD-10 diagnosis, intensity of care need and baseline severity measures)

ICD-10 psychiatric diagnoses assigned by clinicians were used as secondary outcome measures. We used three main categories of diagnoses: F2x (schizophrenia, schizotypal, delusional, and other non-mood psychotic disorders), F3x (mood disorders), or Other/NA (any other diagnosis, or no coded diagnosis). As many patients had more than one recorded diagnosis, we used the ICD-10 hierarchical method to assign one “main diagnosis” per patient, as follows: organic mental disorder > psychotic disorder > mood disorder > anxiety disorder > personality disorder > other psychiatric diagnosis. Presence of a diagnosis in an earlier category trumped diagnosis in subsequent categories, i.e., if a patient had recorded diagnoses of both a psychotic disorder and an anxiety disorder, psychotic disorder was chosen as the main diagnosis.

In addition, we extracted data on intensity of care need, which included the duration of the EIS intervention, the number and duration of inpatient admissions, the number of referrals to crisis and home treatment teams for short-term intensive support in the community, and estimated number of clinical contacts (using number of entries in the EHR as a proxy). We adjusted all intensity of care measures for each patient’s duration of contact with the EIS.

A proportion of the sample had clinical measures of psychiatric illness severity taken at baseline. The specific measure taken varied over time, including the SWEMWBS (Short Warwick Edinburgh Mental Wellbeing Scale, NHS Health Scotland, University of Warwick and University of Edinburgh, 2008, all rights reserved); the Clinical Global Impression (CGI) (Guy, 1976); and the Brief psychiatric rating scale (BPRS) (Faustman and Overall, 1999).

### 2.3 Extraction and coding of variables related to cardiometabolic function and inflammation

Custom-built natural language processing (NLP) software (Cardinal, 2017) was used to extract numerical cardiometabolic and inflammatory marker data from unstructured text, e.g. medical notes. Blood results were included only if they were recorded around the start date of the EIS involvement (±100 days), and if more than one test was recorded within this time frame, the closest to the EIS start date was chosen.

#### 2.3.1 Cardiometabolic biomarkers

Cardiometabolic markers were available for a variable proportion of the sample within the first 100 days from EIS involvement start, and included systolic/diastolic blood pressure (mm/Hg), body mass index (BMI) (kg/m^2^), glucose (mmol/L), glycated haemoglobin (HbA1c) (mmol/mol), and a full lipid profile (total cholesterol (mmol/L) and triglycerides (mmol/L)). Some of the NLP tools for the extraction of cardiometabolic marker data were developed specifically for this study. Accuracy and reliability for all cardiometabolic markers were satisfactory, as measured by *recall* (probability of retrieving a record given it was relevant; >0.75 for all) and *precision* (probability of a record being relevant, given it was retrieved; >0.90 for all) statistics (see (Osimo et al., 2018) for how these were calculated). High-density lipoprotein (HDL) cholesterol levels were not always recorded, so we used the Friedewald equation (Tremblay et al., 2004) to derive this parameter from other lipid levels in patients lacking a direct measure.

#### 2.3.2 Inflammatory markers

We extracted measures of CRP and differential cell counts using methods described previously (Osimo et al., 2018). In keeping with previous analyses and data availability, CRP levels were categorised as follows: ≤3 mg/L (“non-inflamed”); >3 CRP ≤ 10 mg/L (“low-grade inflammation”); >10 mg/L (“suspected infection”). See also the Supplementary Methods.

In addition, we calculated NLR and basophil to lymphocyte (BLR) ratios.

### 2.4 Statistical analysis

*P*-values were adjusted using the Benjamini & Hochberg method. The false-discovery rate (q) was set to 0.05, and results were considered significant when corrected *P-*values were < 0.05. All statistical analyses were performed in R (R Core Team, 2017). Plots were made using ggplot2 (Wickham, 2009), using the Cairo R graphics device (Urbanek and Horner, 2005).

#### 2.4.1 Primary analysis

Cardiometabolic and inflammatory markers at baseline were described by subsequent clinical outcome and diagnosis. An *ANOVA* test was used to compare mean values between groups when data approximated a normal distribution; a Kruskal-Wallis rank sum test was used for skewed continuous data which could not be normalised with log-transformation.

Logistic regression was used to calculate unadjusted odds ratios (ORs) and 95% confidence intervals (95% CIs) for all inflammatory and metabolic markers for participants with poor, compared with good, clinical outcome/illness course. For regression, due to the very skewed nature of some of the variables, all continuous variables (inflammatory and cardio-metabolic markers) were converted into tertiles; the reference category was set to the lowest tertile (≤33th centile). ORs represent the increase in risk for poor outcome/course for participants for each tertile, with the bottom tertile as the reference group.

To examine whether and how confounding may have influenced logistic regression estimates, we performed adjusted analyses in several stages. First, we adjusted for sociodemographic factors (age; sex; ethnicity). Second, we adjusted for BMI. Finally, we conducted a ‘full’ model including all adjustments in the same model.

#### 2.4.2 Sensitivity analyses

Intensity of care need measures were used to validate illness course clinical outcomes. Principal components analysis (PCA) was used to identify the major dimensions of variation and covariation over immune cell counts, using data from all participants. Correlations between variables significantly associated with the primary outcome were explored using the Pearson correlation coefficient and test.

## 3 Results

### 3.1 Sample description

**Fig. 1:**
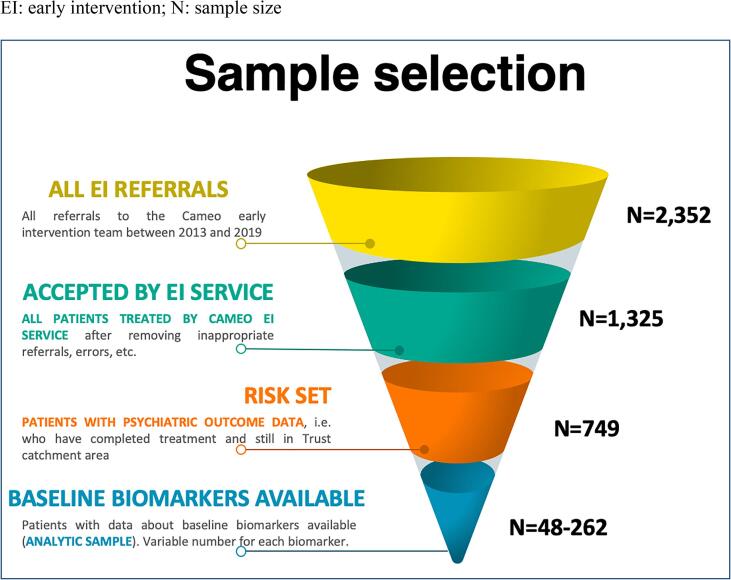
Sample selection. EI: early intervention; N: sample size.

A total of 2352 patients were referred to the CAMEO EIS between January 2013 and November 2019, of which 1325 patients were taken on by the service after excluding inappropriate referrals and technical errors. Of these, 749 had completed EIS involvement, i.e they had not moved our of area and were subsequently discharged either to the care of their GP, or an onward referral was made to another mental health team for continued support. This sample formed the basis for our analysis of long-term clinical outcomes, i.e. the risk set (see Fig. 1). The median duration of EIS involvement for these patients was 655 days (~1.8 years; interquartile range: 246–797 days). Out of the risk set, data on baseline blood biomarkers were available for up to 262 patients (analytic sample). See Table 1 for the baseline socio-demographic characteristics of the sample.

**Table 1:** Sociodemographic characteristics and diagnosis of patients treated by the early intervention service.

### 3.2 Clinical outcome/illness course and intensity of care needs

Out of 749 FEP patients who had completed EIS involvement, 447 patients (59.68%) were discharged to the care of their GP and did not require any secondary care input following discharge from the EI service, indicating either remission or a stable condition (good clinical outcome/ illness course), while 302 (40.32%) needed support from other secondary mental health teams indicating poor clinical outcome/illness course, such as a relapsing/remitting illness or treatment resistance (including treatment with clozapine). We found no evidence that these groups differed in terms of demographic characteristics or ICD-10 psychiatric diagnosis (see Table 1).

With regards to intensity of care measures taken during EIS involvement, patients with poor outcome had a significantly longer duration of EIS involvement (adjusted *P* = 0.01), higher clinical contact rate (adjusted *P* < 0.0001), higher EIS-duration-adjusted number of hospitalisations, higher number of days in hospital (all adjusted *P* < 0.0001), and higher number of high-intensity treatment episodes under crisis and home treatment teams (adjusted *P* < 0.05), compared with those with a good outcome (see Supplementary Table 1).

A proportion of the patients had illness severity measures taken at baseline. Overall, illness severity scores did not differ between patients showing good or poor outcome at discharge (see Supplementary Table 2).

### 3.3 Associations between baseline cardiometabolic measures and clinical outcome/ illness course and psychiatric diagnosis at follow-up

**Fig. 2:**
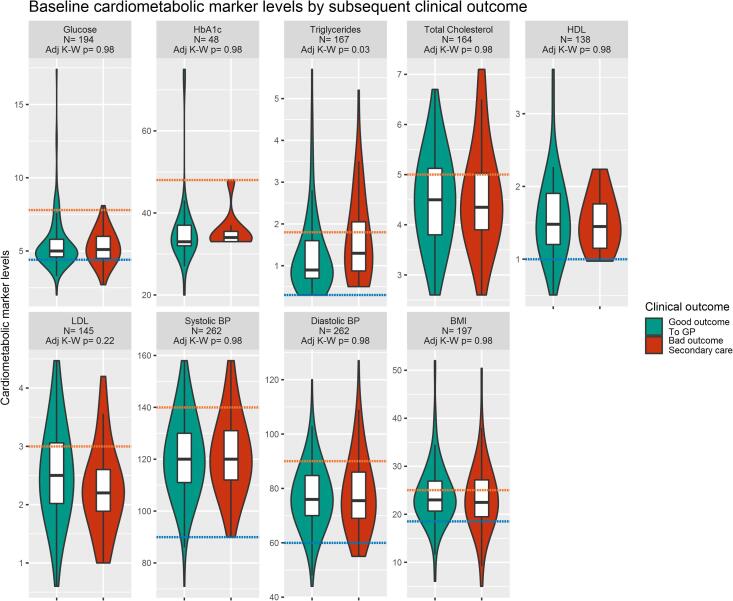
Baseline cardio-metabolic marker measures in groups with good and poor clinical outcome/ illness course at follow-up. Legend: The figure describes baseline cardio-metabolic marker measures in the EI sample by clinical outcome. Boxplots show median and interquartile range, with the outer violin shape showing the density distribution. Discharge to primary care represents a good outcome (teal); requiring specialist care upon discharge represents a worse outcome (red). The number of patients (N) is indicated for each marker. The orange dotted line represents the higher reference range value, the blue dotted line the lower reference range value for each marker. (For interpretation of the references to colour in this figure legend, the reader is referred to the web version of this article.)

Table 2 and Fig. 2 describe the distribution of cardiometabolic measures at baseline by subsequent clinical outcome. Higher baseline triglyceride levels were significantly associated with poor outcome/ illness course at follow-up (*P* = 0.004; BH-adjusted *P* = 0.03). Similarly, logistic regression analyses showed evidence for an association between higher baseline triglycerides levels and poor clinical outcome/illness course at follow-up. The unadjusted OR for poor outcome for those in the top, compared with bottom, tertile of triglycerides levels at baseline was 2.96 (95% CI, 1.32–6.93). Evidence for this association remained after adjusting for potential confounders (adjusted OR = 7.32; 95%CI, 2.26–28.06); see Table 4. However, other baseline cardiometabolic measures were not associated with the subsequent clinical outcome at follow-up (Table 2, Table 4 and Fig. 2). Baseline cardiometabolic markers were not associated with any specific psychiatric diagnosis at follow-up (Supplementary Fig. 1).

**Table 2:** Baseline biochemical and cardio-metabolic factors in patients under the early intervention service (EIS) and in groups with good or poor clinical outcome/ illness course on discharge from EIS.

Legend: **BH-adjusted***P*: *P* adjusted for multiple comparisons using the Benjamini and Hochberg method

*significant result (P < 0.05); ** significant result (P < 0.01).

Differences were tested using Kruskal-Wallis rank sum tests, and the P values shown are already adjusted for multiple comparisons using the BH method. HbA1c: glycated haemoglobin; HDL: high-density lipoprotein; LDL: low-density lipoprotein; BP: blood pressure; BMI: body mass index.

### 3.4 Associations between baseline differential cell counts and clinical outcome/ illness course and psychiatric diagnosis at follow-up

**Fig. 3:**
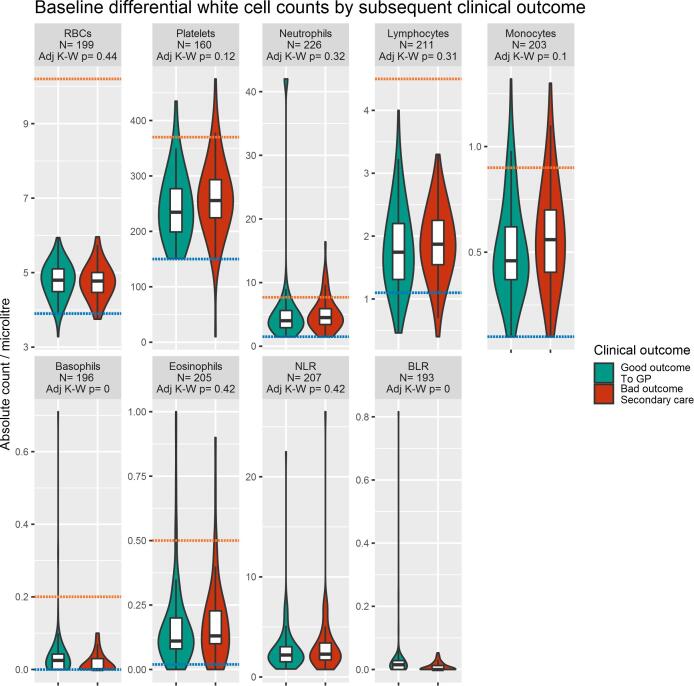
Baseline differential cell counts in groups with good and poor clinical outcome/ illness course at follow-up. Legend: The figure shows a comparison between cell counts in the EI sample by clinical outcome. Boxplots show median and interquartile range, with the outer violin shape showing the full distribution of data. Discharge to primary care represents a good outcome (teal); requiring specialist care upon discharge represents a worse outcome (red). The number of patients (N) is indicated for each marker. The orange dotted line represents the higher reference range value, the blue dotted line the lower reference range value for each marker. Differences were tested using Kruskal-Wallis rank sum tests, and the p values shown are already adjusted for multiple comparisons using the BH method. NLR: neutrophil to lymphocyte ratio; BLR: basophil to lymphocyte ratio. (For interpretation of the references to colour in this figure legend, the reader is referred to the web version of this article.)

Table 3 and Fig. 3 describe the distribution of differential cell counts at baseline by subsequent clinical outcome. Lower baseline basophil count and BLR were significantly associated with poor clinical outcome/ illness course (BH-adjusted *P* = 0.001 and *P* < 0.001, respectively). Baseline monocyte counts were higher in the group with poor outcome; however, this was not significant after correcting the *P-*value for multiple testing.

**Table 3:** Baseline differential cell counts in patients under the early intervention service (EIS) and in groups with good or poor clinical outcome/ illness course on discharge from EIS.

Legend: **BH-adjusted** P: P adjusted for multiple comparisons using the Benjamini and Hochberg method

*significant result (P < 0.05); ** significant result (P < 0.01).

**Table 4:** Odds ratios (95% CI) for poor clinical outcome/illness course on discharge from the early intervention service associated with baseline biochemical, cardio-metabolic and inflammatory measures.

Predictor	N with data	Measure Tertile (Cut-off values) [reference = bottom tertile]	Odds ratios (95% CI) for poor clinical outcome/illness course			
M			Unadjusted Model	Adjusted for age, sex, ethnicity	Adjusted for BMI	Adjusted for age, sex, ethnicity, BMI
Random glucose (mmol/l)	194	Middle (>4.7, ≤5.4)	0.65 (0.30–1.39)	0.65 (0.30–1.39)	0.56 (0.16–1.87)	0.54 (0.15–1.87)

Top (>5.4)	1.07 (0.52–2.23)	1.06 (0.50–2.25)	1.29 (0.40–4.25)	1.50 (0.43–5.42)

HbA1c (mmol/l)	48	Middle (>33, ≤36)	1.31 (0.16–8.23)	1.13 (0.12–8.27)	N/A*	N/A*

Top (>36)	0.95 (0.12–5.74)	1.99 (0.17–19.24)	N/A*	N/A*

Triglycerides (mmol/l)	167	Middle (>0.8, ≤1.4)	1.58 (0.64–3.91)	1.62 (0.66–4.02)	2.15 (0.53–8.77)	2.12 (0.52–8.72)

Top > 1.4	**2.96 (1.32**–**6.93)**	**3.26 (1.42**–**7.86)**	**6.06 (2.03**–**21.02)**	**7.32 (2.26**–**28.06)**

Total cholesterol (mmol/l)	164	Middle (>4, ≤4.9)	1.02 (0.46–2.29)	0.99 (0.44–2.24)	1.51 (0.50–4.81)	1.47 (0.48–4.74)

Top (>4.9)	0.84 (0.35–1.98)	0.82 (0.33–2.02)	1.65 (0.47–5.97)	1.64 (0.44–6.30)

HDL (mmol/l)	138	Middle (>1.22, ≤1.63)	1.36 (0.55–3.43)	1.30 (0.52–3.32)	1.84 (0.54–6.88)	1.87 (0.54–7.09)

Top (>1.63)	0.88 (0.34–2.30)	0.86 (0.32–2.27)	1.07 (0.29–4.19)	1.04 (0.28–4.19)

LDL (mmol/l)	145	Middle (>2.04, ≤2.73)	1.29 (0.56–3.03)	1.26 (0.55–2.96)	0.91 (0.27–3.11)	0.89 (0.26–3.09)

Top (>2.73)	0.41 (0.15–1.09)	0.40 (0.14–1.08)	0.81 (0.24–2.69)	0.80 (0.23–2.68)

Systolic BP (mmHg)	262	Middle (>115, ≤127)	1.00 (0.55–1.82)	0.97 (0.53–1.78)	1.08 (0.46–2.55)	1.07 (0.44–2.59)

Top (>127)	0.98 (0.53–1.81)	1.02 (0.54–1.95)	1.28 (0.56–2.96)	1.42 (0.57–3.59)

Diastolic BP (mmHg)	262	Middle (>72, ≤82)	0.75 (0.41–1.36)	0.81 (0.44–1.50)	1.24 (0.54–2.86)	1.40 (0.60–3.32)

Top (>82)	0.95 (0.51–1.74)	1.08 (0.57–2.06)	1.19 (0.51–2.81)	1.37 (0.57–3.36)

BMI (kg/m^2^)	197	Middle (>21.4, ≤25)	0.71 (0.33–1.51)	0.84 (0.38–1.86)	N/A**	N/A**

Top (>25)	0.85 (0.41–1.75)	0.90 (0.43–1.88)	N/A**	N/A**

Red blood cells (RBC) count	199	Middle (>4.6, ≤5)	1.12 (0.56–2.24)	1.21 (0.59–2.52)	1.15 (0.41–3.24)	1.08 (0.36–3.21)

Top (>5)	0.65 (0.31–1.36)	0.77 (0.34–1.74)	0.75 (0.24–2.28)	0.67 (0.18–2.45)

Platelets count	160	Middle (>216, ≤271)	2.00 (0.91–4.52)	1.85 (0.82–4.23)	0.95 (0.30–3.05)	0.94 (0.29–3.04)

top (>271)	**2.88 (1.29**–**6.63)**	**2.63 (1.16**–**6.16)**	2.85 (0.92–9.35)	3.09 (0.97–10.50)

Neutrophils count	226	Middle (>3.44, ≤5.05)	1.84 (0.92–3.77)	1.82 (0.89–3.78)	1.84 (0.65–5.40)	1.90 (0.66–5.65)

Top (>5.05)	1.65 (0.83–3.33)	1.49 (0.73–3.08)	2.37 (0.85–6.91)	2.42 (0.83–7.44)

Lymphocytes count	211	Middle (>1.47, ≤2.06)	**2.23 (1.09**–**4.73)**	**2.39 (1.15**–**5.15)**	**3.35 (1.13**–**11.42)**	**3.49 (1.15**–**12.11)**

Top (>2.06)	1.70 (0.80–3.72)	1.79 (0.83–3.95)	2.30 (0.71–8.28)	2.41 (0.73–8.77)

Monocytes count	203	Middle (>0.4, ≤0.57)	1.55 (0.72–3.38)	1.52 (0.70–3.34)	0.96 (0.26–3.29)	0.91 (0.24–3.15)

Top (>0.57)	**2.33 (1.17**–**4.75)**	**2.35 (1.17**–**4.82)**	**2.74 (1.01**–**7.88)**	**2.78 (1.02**–**8.06)**

Basophils count	196	Middle (>0, ≤0.04)	**0.32 (0.16**–**0.64)**	**0.31 (0.15**–**0.61)**	**0.26 (0.08**–**0.76)**	**0.24 (0.07**–**0.72)**

Top (>0.04)	**0.27 (0.10**–**0.62)**	**0.25 (0.10**–**0.60)**	0.47 (0.12–1.56)	0.40 (0.10–1.39)

Eosinophils count	205	Middle (>0.1, ≤0.2)	0.66 (0.32–1.33)	0.72 (0.35–1.47)	0.75 (0.25–2.06)	0.77 (0.26–2.16)

Top (>0.2)	1.29 (0.62–2.63)	1.43 (0.68–3.00)	1.34 (0.43–3.91)	1.41 (0.44–4.29)

Neutrophil to lymphocyte ratio (NLR)	207	Middle (>1.86, ≤2.9)	1.50 (0.76–3.03)	1.41 (0.70–2.88)	1.50 (0.54–4.30)	1.45 (0.51–4.20)

Top (>2.9)	1.25 (0.60–2.60)	1.15 (0.54–2.47)	1.44 (0.49–4.30)	1.42 (0.46–4.45)

Basophil to lymphocyte ratio (BLR)	193	Middle (>0, ≤0.02)	**0.44 (0.21**–**0.90)**	**0.41 (0.19**–**0.85)**	0.48 (0.16–1.33)	0.43 (0.14–1.22)

Top (>0.02)	**0.19 (0.07**–**0.43)**	**0.19 (0.07**–**0.43)**	**0.16 (0.02**–**0.65)**	**0.14 (0.02**–**0.58)**

CRP	52	4-10mg/L	1.50 (0.37–6.17)	1.72 (0.35–8.78)	N/A*	N/A*
		>10mg/L	1.78 (0.48–6.83)	2.30 (0.55–10.44)	N/A*	N/A*

Note: All values were divided into tertiles. Values are the OR for the middle or top tertile over the reference (bottom) tertile for each variable. In bold are significant results

bold face means values are significantly different from OR of 1 (no difference)

* insufficient sample with all independent variables available

** BMI cannot be both independent variable and predictor

Logistic regression analyses showed evidence for an association between multiple baseline cell counts and poor clinical outcome/ illness course at follow-up, including for BLR, monocytes, basophils, lymphocytes and platelets (Table 4). For BLR, the unadjusted OR for poor outcome for those in the top, as compared with those in the bottom tertile was 0.19 (95% CI, 0.07–0.43), which remained significant after adjusting for confounders (adjusted OR = 0.14; 95% CI, 0.02–0.58). For monocytes, the adjusted OR for poor outcome for those in the top, as compared with those in the bottom tertile, was 2.78 (95% CI, 1.02–8.06). For basophils, the adjusted OR for poor outcome for those in the middle, as compared with those in the bottom tertile was 0.24 (95% CI, 0.07–0.72). For lymphocytes, the adjusted OR for poor outcome for those in the middle, as compared with those in the bottom tertile, was 3.49 (95% CI, 1.15–12.11). For platelets, the unadjusted OR for poor outcome for those in the top, as compared with those in the bottom tertile was 2.88 (95% CI, 1.29–6.63), but it became non-significant after adjusting for BMI. Other baseline cell counts were not associated with subsequent clinical outcomes at follow-up (Table 3, Table 4 and Fig. 3).

With regards to the associations between baseline cell counts and subsequent psychiatric diagnoses, Supplementary Fig. 2 shows that lymphocyte counts varied significantly between diagnostic groups, with higher counts in primary psychosis compared with mood disorders (overall BH-adjusted *P* = 0.02). Baseline NLR also differed between subsequent diagnoses: NLR was higher in patients diagnosed with a mood disorder or psychosis as compared to those with other/no available diagnoses (overall BH-adjusted *P* = 0.03), but no significant difference was present directly comparing patients diagnosed with a mood disorder and psychosis (*P* = 0.36). Other baseline cell counts were not significantly different between diagnostic groups.

Many of the inflammatory measures were correlated, so we also summarised cell count data using principal components analysis. Principal components 1 and 2 of a PCA of baseline differential cell counts were not significantly different by subsequent diagnosis or illness course; more details are described in the Supplementary results.

#### 3.4.1 Correlation between cardiometabolic and inflammatory measures

Correlations between baseline cardiometabolic and inflammatory measures were explored for the sample. These can be found as Supplementary Fig. 3.

### 3.5 Associations between baseline CRP levels and clinical outcome/ illness course and psychiatric diagnosis at follow-up

Data on both baseline plasma CRP levels and subsequent clinical outcome/illness course were available for 52 patients only. Of these, 14 (26.92%) showed evidence of low-grade inflammation (CRP > 3 mg/L but ≤ 10 mg/L), and 17 (32.69%) had evidence of suspected infection (CRP > 10 mg/L). The prevalence of low-grade inflammation (CRP 3–10 mg/L) at baseline was somewhat higher in the group with subsequent poor clinical outcome/illness course (28.57%) than in those with a good outcome (25.81%); however, this difference was not statistically significant (2-sided Fisher’s exact test *P* = 0.68). Baseline inflammation was not significantly associated with any specific diagnosis subsequently at follow-up (*P* = 0.36).

Logistic regression analyses showed no evidence for an association between baseline levels of CRP and poor clinical outcome/ illness course at follow-up. Table 4 shows that the unadjusted OR for poor outcome for patients with CRP > 10 mg/L, as compared to those with CRP ≤ 3 mg/L, was 1.78 (95% CI, 0.48–6.83). ORs adjusted by BMI could not be calculated due to a small sample size.

## 4 Discussion

Using real-world clinical data from electronic health records (EHR) from an EI service in England, we examined clinical outcomes/illness course and intensity of care need in a sample of patients with FEP. The majority of patients with FEP had favourable long-term clinical outcomes, as ~60% were discharged to primary care with no ongoing involvement of secondary mental health services.

We tested for associations between blood cardiometabolic and inflammatory markers at baseline and clinical outcomes at follow-up. Higher triglyceride levels were associated with a higher risk of a subsequent poor clinical outcome/ illness course. We also found that higher monocyte, lymphocyte and platelet counts were directly associated with a subsequent poor clinical outcome/illness course. Basophil counts and BLR were protective. Some of these ORs did not consistently reach the significance threshold for all levels of adjustment which could be due to limited sample size. Other baseline measures, including glucose, blood pressure, BMI, cholesterol levels, other cell counts, CRP levels and the first two principal components of differential cell counts were not significantly associated with psychiatric illness course. In terms of diagnosis, lymphocyte counts were significantly lower, and NLR higher, in patients subsequently diagnosed with a primary mood-disorder related psychosis (unipolar depression or bipolar disorder).

To our knowledge, this is the first study measuring longitudinal associations between 4 demographic measures, 10 immune, and 9 metabolic baseline predictors and long-term psychiatric outcomes including diagnosis and illness course in a large sample of patients with FEP. Our results suggest that multiple blood-based markers may have a prognostic potential for patients with a FEP. Several previous cross-sectional studies measured immune and metabolic measures in FEP. However, only a handful of longitudinal studies exist that have measured markers at baseline and psychiatric outcomes at follow-up in FEP. These tended to be studies involving a small cohort followed up for up to one year (Mondelli et al., 2015, Nettis et al., 2019). In this study we were able to harness EHR data to extract baseline data – originally collected for clinical reasons – and use it in conjunction with diagnosis and team referral data to study associations with long-term clinical outcomes in a much larger cohort of 749 FEP patients.

In this study we classified clinical outcome/illness course using a binary descriptor based on whether FEP patients needed further secondary care support following discharge from the EIS (poor clinical outcome/ illness course) or not (good outcome). These two groups were not significantly different in terms of psychiatric diagnosis, sociodemographic characteristics and baseline severity of illness. However, patients with a poor outcome had higher intensity of care needs during their involvement with EIS as reflected by higher number of clinical contacts, hospitalisations and number of high-intensity crisis and home treatment team support, indicating that discharge destination is a useful proxy for prognosis.

Our study does not seek to establish whether these associations represent causal relationships, but to document associations, which could inform future research into causes, interventions or prognostics. Our study leaves open the possibility that some of the documented associations could be causal, and therefore potential treatment targets, or epiphenomenal, reflecting the fact that patients with worse prognosis have other biological or social associations that predispose them to differential baseline biomarker levels. Irrespective of whether or not the relationships are causal, biomarker associations with important clinical outcomes could, if replicated, potentially contribute to clinical care as prognostic markers.

Our findings of an association between elevated triglyceride levels and risk of a worse outcome are compatible with a previous study of 42 FEP patients, which found that a principal component factor including immune and metabolic factors (CRP, triglyceride levels and BMI) was related to worse outcomes (higher PANSS scores) at about one year (Nettis et al., 2019). Previous cross-sectional evidence has also consistently associated FEP with higher triglyceride levels, without being able to correlate the finding with illness course (Perry et al., 2018, Pillinger et al., 2017). It is therefore likely that there is an association between higher triglycerides and FEP, and in this study we also find – within FEP – an association between higher triglycerides and a more severe illness course. It is unclear how triglyceride levels could influence psychiatric clinical outcomes, but our results show that common demographic factors or BMI are unlikely to be sole mediators of this association. Triglycerides, which are known to play a role in the development of insulin resistance (Banks et al., 2018), might warrant consideration as a potential biological link between schizophrenia and cardiometabolic disease, which may be genetic (Lin and Shuldiner, 2010).

We also found evidence of an inverse association between basophil counts/BLR and a worse psychiatric illness course. Furthermore, our sensitivity analyses show that the association with BLR was not fully explained by demographic factors or BMI. For basophils, the adjustment for BMI attenuated the association and 95% CI included the null, but this could be due to a reduction in sample size from 195 in unadjusted to 109 in BMI-adjusted analyses. It is also interesting to note that basophil counts did not correlate with any other measures, and could therefore explain a different portion of the variance from other measures. This finding is new, and, to our knowledge, basophil counts have not been previously studied in relation to clinical outcomes in FEP. Two previous cross-sectional studies comparing basophil counts in FEP with healthy controls showed no difference in the two groups (Jackson and Miller, 2019). BLR is a somewhat under-researched measure, and a low BLR has previously been described as associated with most systemic autoimmune rheumatic diseases (Yang et al., 2017); no associations have been made with psychiatric illness before.

Both basophil counts and triglyceride levels appear to be involved in the insulin resistance and diabetes pathways (Harsunen et al., 2013, Koyama et al., 1997, Lee et al., 2014), and, as discussed earlier, there are promising associations between insulin resistance/diabetes and psychosis. Basophil counts should be investigated further in relation to psychosis, and with a particular outlook on illness course.

We also found variable degrees of evidence for associations between baseline monocyte, lymphocyte and platelet counts and a worse psychiatric outcome, although platelets and lymphocyte associations became non-significant in adjusted analyses. These three cell counts were strongly correlated. Monocytes are known to be elevated in FEP, while lymphocytes were found to be non-significantly elevated in previous research (Jackson and Miller, 2019). However, Mendelian randomisation analyses have shown evidence for potential a causal association between higher lymphocyte counts and schizophrenia (Astle et al., 2016). We add to this evidence by reporting of the potential value of measuring lymphocytes as a prognostic marker in FEP.

Finally, in this study we did not find a significant association between elevated CRP and psychotic illness course. This result is perhaps surprising since there is a wealth of cross sectional (Miller et al., 2014, Pillinger et al., 2019) and longitudinal (Khandaker et al., 2014) evidence for an association between elevated CRP and psychosis, including with prognosis (Nettis et al., 2019). However, our findings may also be explained by the limited power in our CRP analysis due to data unavailability, as discussed in the limitations section below.

Furthermore, this study did not find a significant association between BMI and psychiatric illness course, in contrast to Nettis and colleagues (Nettis et al., 2019). Previous studies have reported associations between BMI and psychosis. Lower BMI in childhood is associated with later development of non-affective psychosis (Sormunen et al., 2018). There is also evidence for a genetic correlation between BMI and schizophrenia (Bulik-Sullivan et al., 2015). Our negative finding could therefore be a type 2 error, or it could a true finding related to specific population characteristics of our cohort.

### 4.1 Strengths and limitations

This work is based on a large sample of 749 FEP patients, with a follow-up of up to 5 years (median of ~2 years). As opposed to previous studies which have recruited patients and assigned psychiatric diagnoses based on one assessment, in this study patients were enrolled in a years-long process of assessment and treatment within a specialised EI in psychosis team, which fosters greater confidence in the psychiatric phenotype for people enrolled in this study. This guarantees a high degree of homogeneity of the sample with regards to the psychiatric presenting syndrome. Another strength of this study, as compared to studies selected for research on specific criteria, is the naturalistic study design, including a large number of consecutive referrals with very little possibility of selection bias. As the only EIS for FEP in Cambridgeshire, our CAMEO sample covers a large proportion of all incident cases of first episode psychosis in a geographically defined catchment area. Further strengths include the use of a large number of biomarkers, including some previously under-studied in FEP and psychosis in general, such as basophils, eosinophils, platelets, and RBCs. Furthermore, we used clinical laboratory measures, so we have a high confidence in the high accuracy and precisions of our measures, and as to their clinical applicability.

A limitation of this work is the lack of antipsychotic medication data at baseline. Antipsychotics could influence the levels of cardiometabolic markers. However, most patients admitted to CAMEO are medication naïve or minimally treated, and only patients who have been taking antipsychotic medication for less than 6 months are accepted by the team. Bloods tests were carried out within 100 days of referral to the team so it’s likely that some patients would have been started on antipsychotic medication during this time, though the duration of treatment is likely to be relatively short.

Among the limitations, this design does mean that a variable sample size is available for each measure, due to clinician ordering decisions, patient refusal rates, and because of differences in lab ordering protocols for newly admitted patients over time. In statistical terms, variable sample sizes for different measures means limited power for multi-variable analyses. More specifically, when applying stepwise adjustment, such as we have done for BMI, adjusted analyses relied on a smaller sample than un-adjusted ones, therefore making it more likely that negative adjusted findings might suffer from a type 2 statistical error. Some evidence for this can be seen in Table 4, where a number of ORs are relatively high, but adjustment causes a widening of the 95%CIs to the point of including the null. Furthermore, only a small proportion of the sample had baseline psychometric information on severity available.

Specifically for CRP analyses we did not have an adequately large sample due to missing data. Consequently, our negative findings with regards to the associations between CRP levels and outcomes could be reflecting a type 2 error due to low power.

Other limitations of a clinical sample are the unavailability of potentially relevant information, such as data about physical activity levels, smoking or alcohol use.

## 5 Conclusions and future directions

In this longitudinal study we report that higher triglyceride levels, higher platelet, monocyte and lymphocyte counts, and lower basophil counts and BLR at baseline are associated with a poor clinical outcome/ illness course in FEP patients in 1–5 years. These findings need replication in other samples, and could inform the development of algorithms for the prediction of prognosis of long-term outcomes in FEP in clinical settings.

## 6 Financial disclosures

The authors have no conflict of interests or financial disclosures to declare.
