# Supplementary material for: Inflammatory and cardiometabolic markers at presentation with first episode psychosis and long-term clinical outcomes: A longitudinal study using electronic health records
Source: Brain Behav Immun. 2021 Jan;91:117–27. doi: 10.1016/j.bbi.2020.09.011 (PMC7773969; doi:10.1016/j.bbi.2020.09.011)
Supplement: Supplementary data 1 [file mmc1.docx]

## Supplementary Methods**:**

### Extraction and Coding of Cardiometabolic and Inflammatory Marker Data

All extracted data was subject to data quality checks, and discarded if the values were unrealistic (e.g. negative) or less than 1/10^th^ times the lower reference range, or more than 10 times the upper reference range for each analyte.

Blood samples from patients in Cambridge or Peterborough were tested in different labs, using assays with different sensitivity; some of these labs used low-sensitivity assays that could only detect CRP levels >10mg/L. We had to exclude data categorised as <10mg/L as not fitting into our categories (≤3 mg/L (non-inflamed); >3ml/L and ≤10 mg/L (low-grade inflammation)).

According to the US Centers for Disease Control and Prevention and American Heart Association guidelines CRP levels over 3mg/L is considered to be high (Pearson et al., 2003; Ridker, 2003); such levels are associated with increased risks of cardiovascular disease (Koenig et al., 1999) and psychiatric illnesses such as schizophrenia (Metcalf et al., 2017) in population-based studies. For the purpose of this study, we have defined low-grade inflammation as a serum CRP level >3mg/L. This is because the hospital laboratory only reported an exact value for CRP if it was equal or over 4mg/L; levels below this threshold were reported as ≤3mg/L.

## Supplementary results

### Correlations and PCA of cell counts

We estimated correlations between cell counts in the sub-sample of patients with no missing data for white cell count (N=359). Significant correlations (p<0.01 to account for multiple testing) existed between lymphocytes and monocytes, neutrophils and eosinophils; neutrophil counts correlated with monocytes; basophil and eosinophil counts were also correlated (**Supplementary Figure 3**); therefore, we used principal components analysis (PCA) to summarize cell counts in terms of two orthogonal, linear combinations of the 5 measures which accounted for the greatest proportion of the variance-covariance. The first principal component (PC1), which accounted for 31.3% of the total variance-covariance, was a weighted average of all counts. The second principal component (PC2) accounted for 23.6% of the total variance-covariance and showed a direct correlation with lymphocyte, basophil and eosinophil counts, while also showing an inverse relationship to all other counts (**Supplementary figure 4**).

**Supplementary figures 5 and 6** describe the distribution of PC1 and PC2 of cell counts at baseline by subsequent clinical outcome, clinical course and diagnosis respectively. There were no significant associations.

## Supplementary Figures

###### Supplementary Figure 1: Baseline cardiometabolic factors by subsequent psychiatric diagnosis:

The figure describes baseline cardio-metabolic marker measures in the EI sample by subsequent diagnosis (primary psychotic disorders, mood disorders, or any other diagnosis and no coded diagnosis). Boxplots show median and interquartile range, with the outer violin shape showing the full distribution of data. Patients discharged to primary care represent a good outcome; patients requiring secondary care input upon discharge represent a worse outcome. The N is indicated for each marker. The red dotted line represents the higher reference range value, the green dotted line the lower reference range value for each marker.

Differences were tested using Kruskal-Wallis rank sum tests, and the p values shown are already adjusted for multiple comparisons using the BH method.

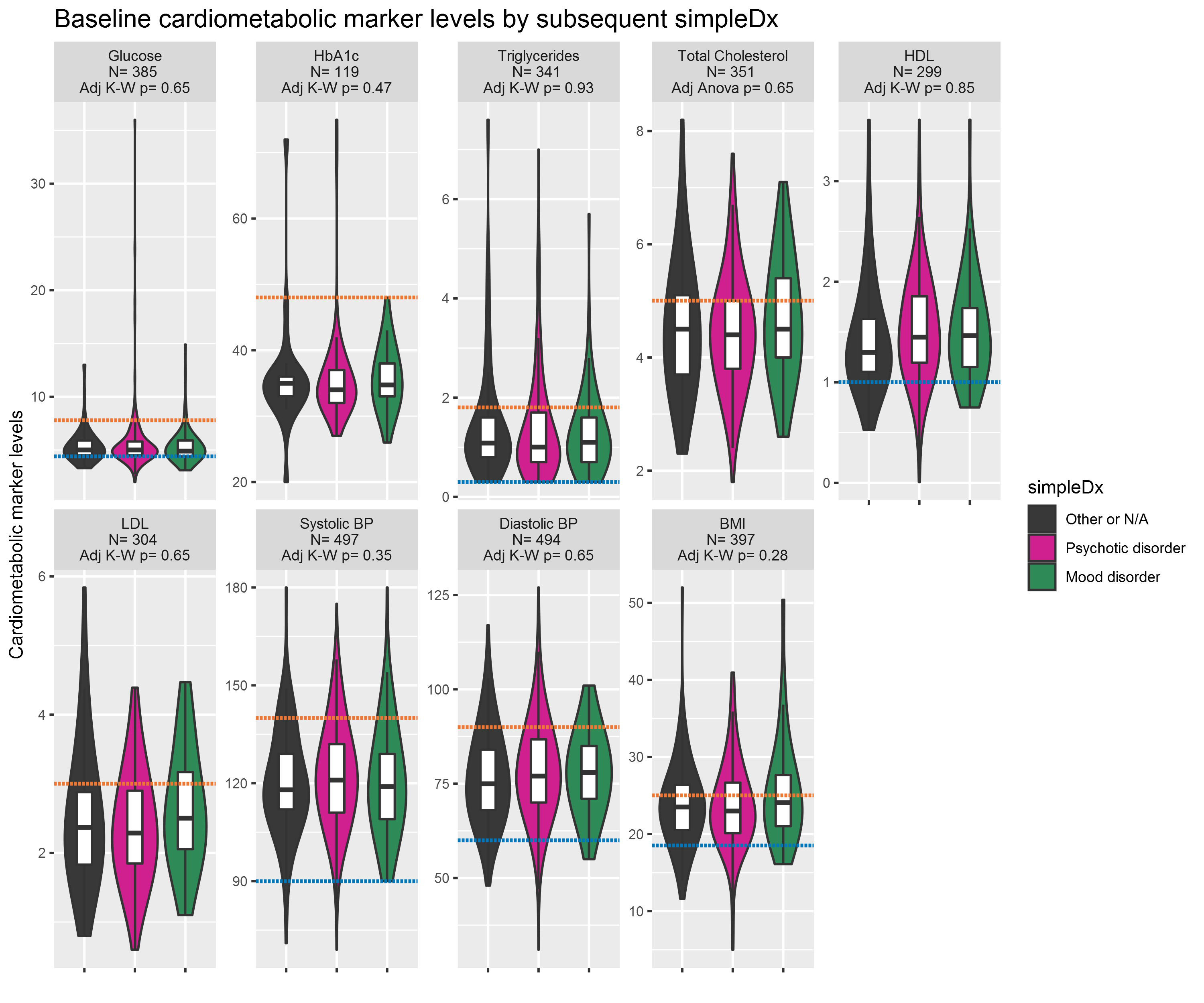


###### Supplementary Figure 2: Baseline differential cell counts by subsequent psychiatric diagnosis:

The figure describes cell counts in the EI sample by subsequent diagnosis (primary psychotic disorders, mood disorders, or any other diagnosis or no coded diagnosis). Boxplots show median and interquartile range, with the outer violin shape showing the full distribution of data. Patients discharged to primary care represent a good outcome; patients requiring secondary care input upon discharge represent a worse outcome. The N is indicated for each marker. The red dotted line represents the higher reference range value, the green dotted line the lower reference range value for each marker.

Differences were tested using Kruskal-Wallis rank sum tests, and the p values shown are already adjusted for multiple comparisons using the BH method. Solid lines and p values in blue indicate variable by variable Kruskal-Wallis rank sum tests; the results for these are not BH-adjusted.


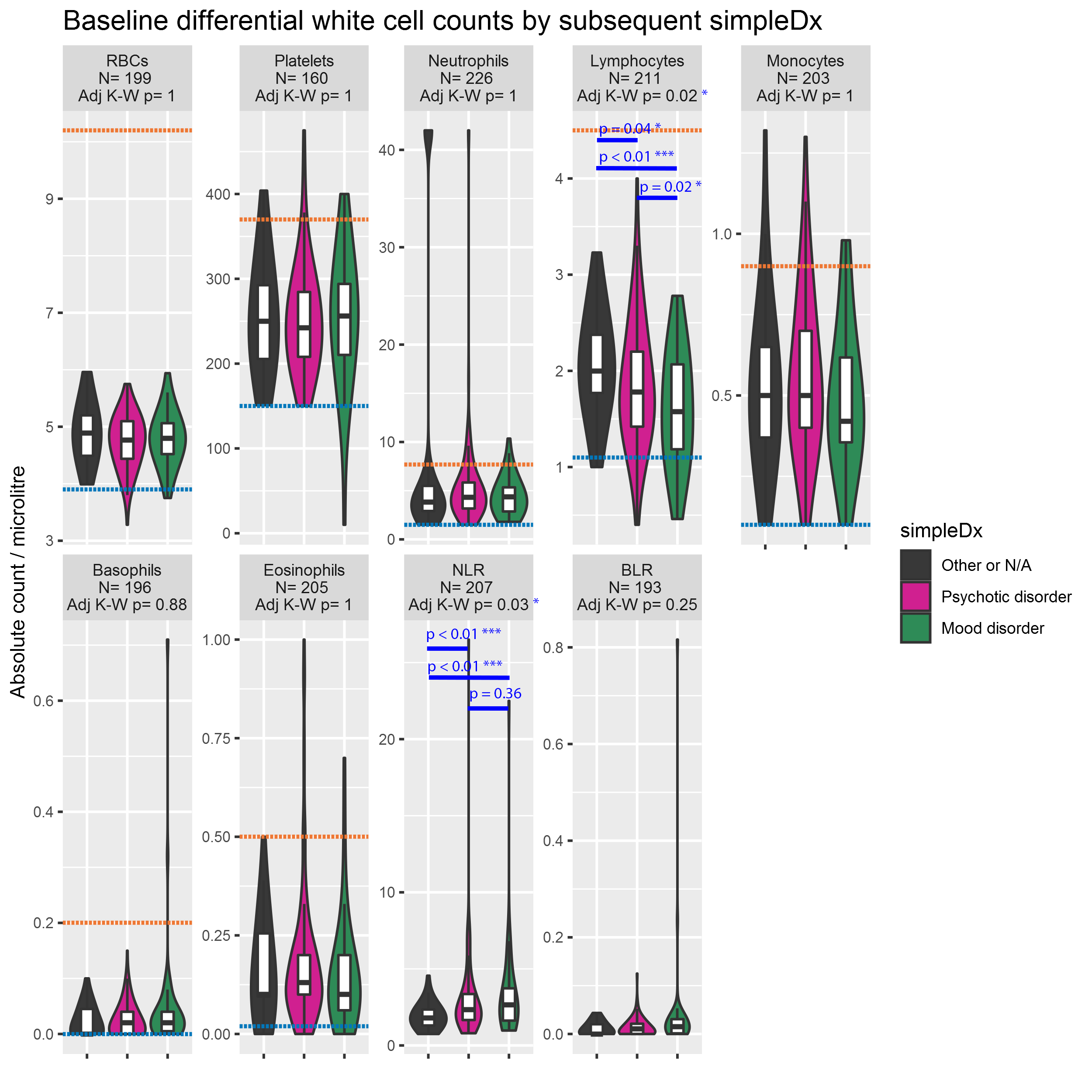


###### Supplementary Fig 3: correlation plot of cardiometabolic and inflammatory measures

The figure shows a matrix of Pearson's correlation coefficient, *r*, for all possible pairs of cardiometabolic and cell count measures. Positive correlations are displayed in blue and negative correlations in red. The colour intensity is proportional to the correlation coefficients. P values were calculated and adjusted for multiple testing using BH, and stars indicate significance (no stars, P>=0.05; one star, <0.05; two stars, <0.01; three stars, <0.001).

######
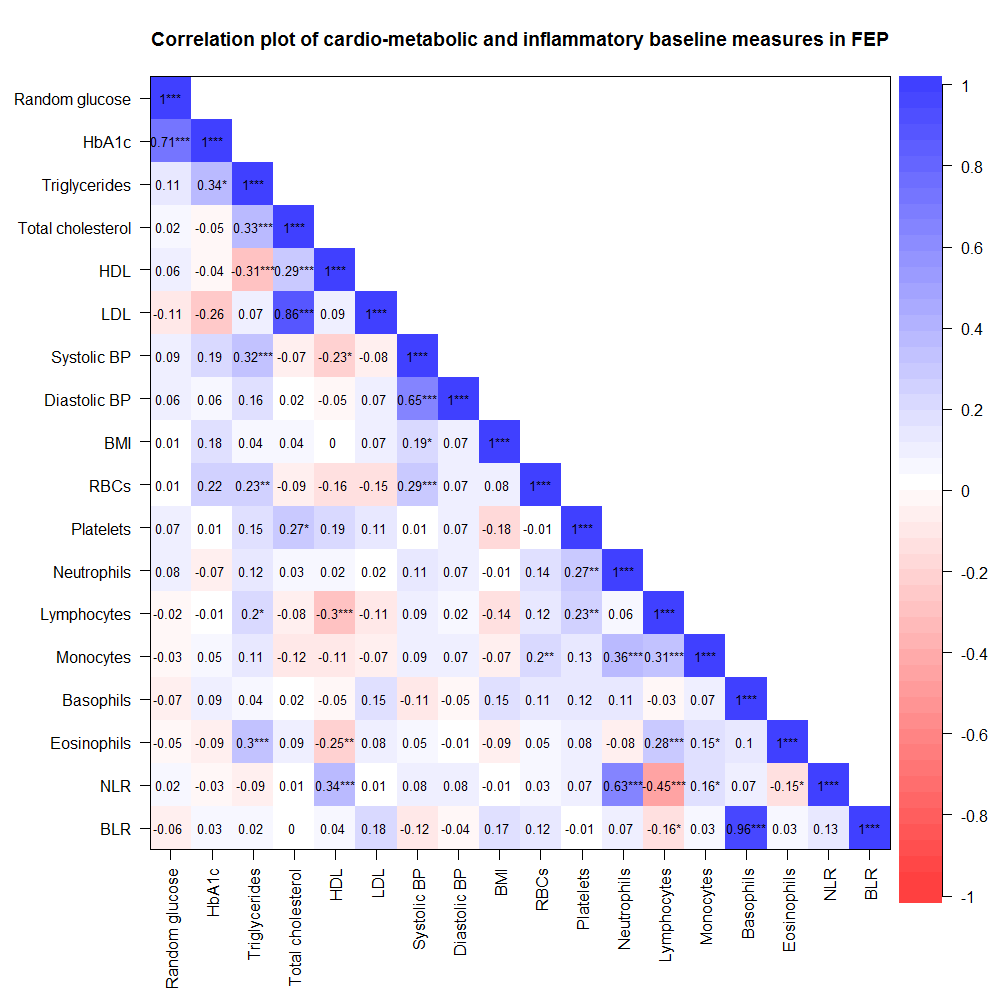


###### Supplementary Fig 4: Principal Component Analysis of cell counts biplot


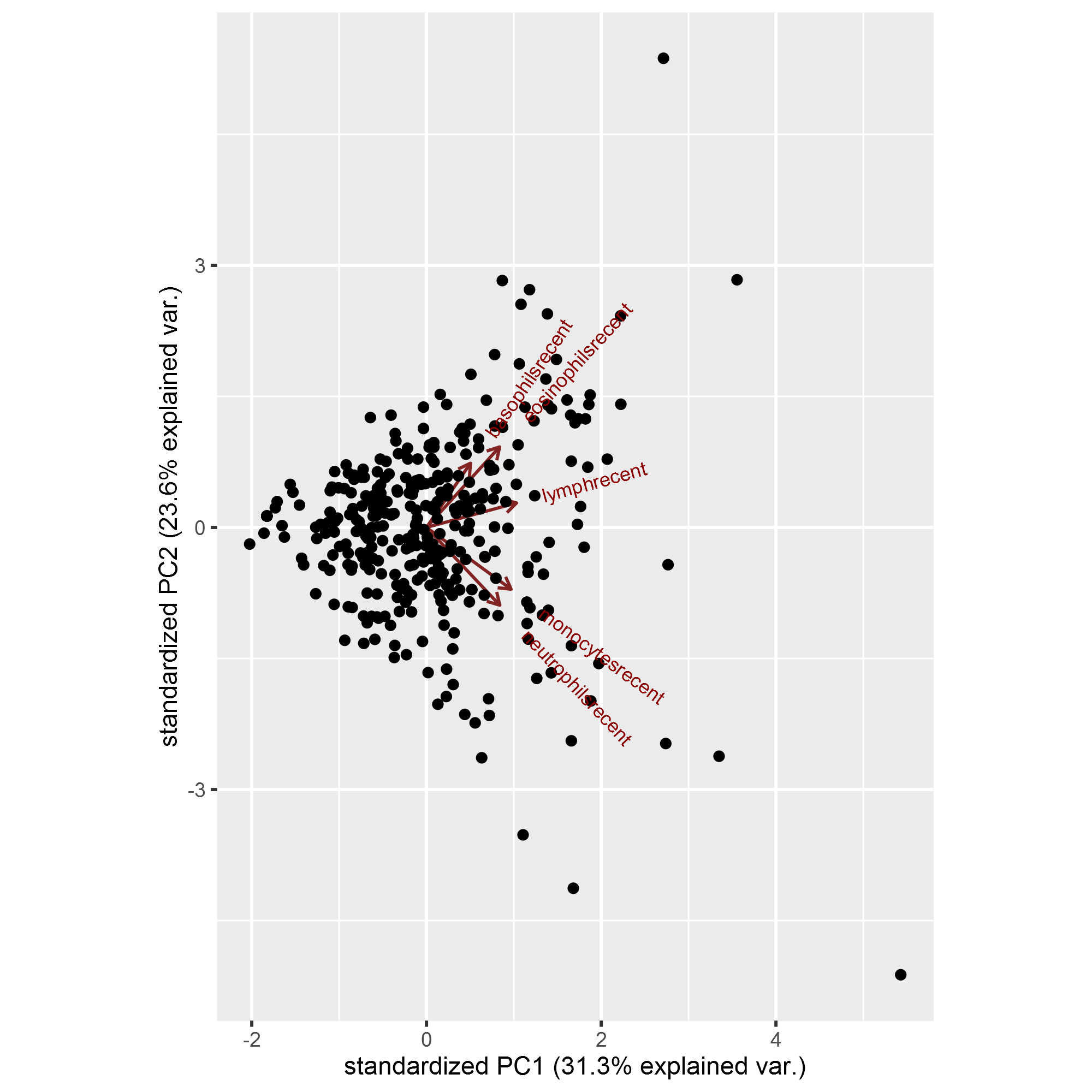


###### Supplementary Fig 5: PC1 and PC2 of cell counts by subsequent clinical outcome

**
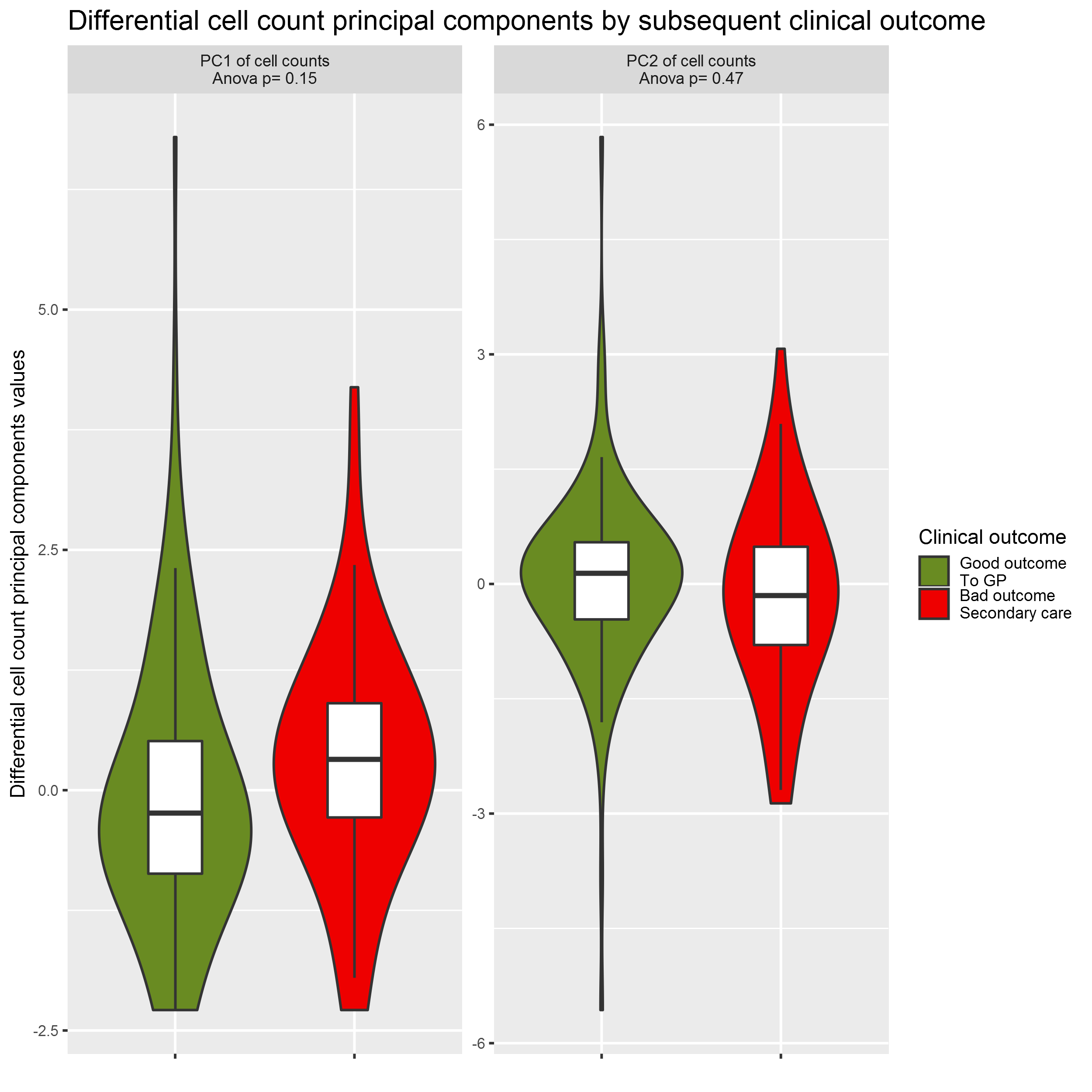
**

###### Supplementary Fig 6: PC1 and PC2 of cell counts by subsequent diagnosis


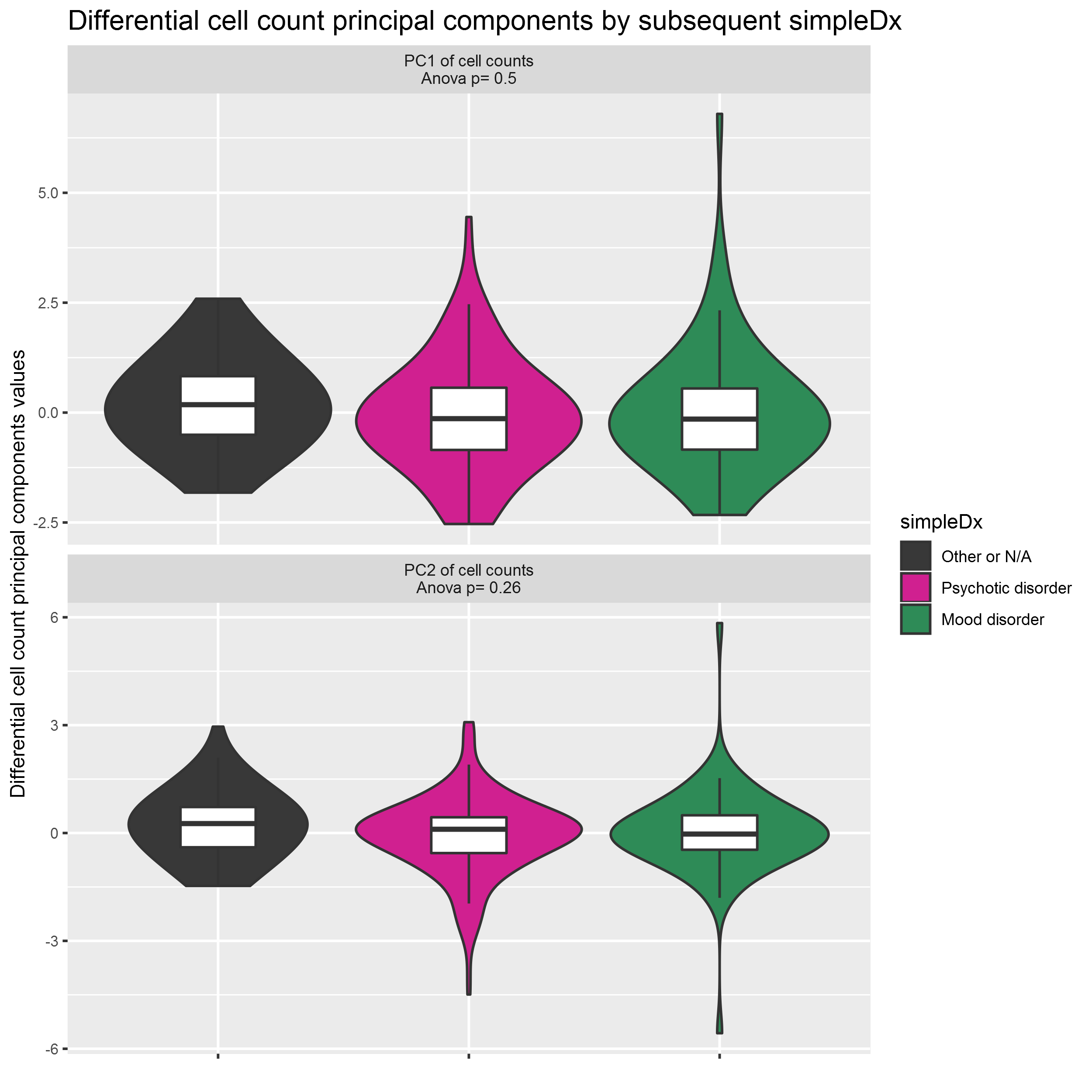


## Supplementary Tables

###### Supplementary Table 1: Intensity of care in patients, overall and by clinical outcome

Some ratios are multiplied by a factor of 2500 to make the number more easily readable

| **Characteristic** | **Sample with longitudinal psychiatric outcomes** | **Primary care upon discharge** | **Secondary psychiatric care upon discharge** | **Difference between primary vs secondary care - test statistic^1^** | **BH adjusted p** |
| --- | --- | --- | --- | --- | --- |
| Sample size | 749 | 447 (59.68%) | 302 (40.32%) |  |  |
| Duration of CAMEO intervention, days, median (25^th^-75^th^ centile) | 655 (246-797) | 540 (214-770) | 734 (297-837) | **Kruskal-Wallis *χ^2^=* 10.3, df = 1, p=0.001** | **0.009** |
| Clinical contact rate (N of clinical contacts/time with CAMEO in days)*2,500, median (25^th^-75^th^ centile) | 618.43 (279.9-1219.51) | 538.83 (272.09-988.39) | 827.13 (329.07-1806.72) | **Kruskal-Wallis *χ^2^=*19.9, df = 1, p<0.0001** | **<0.0001** |
| Inpatient admission rate (N of admissions/time with CAMEO in days)*2,500, median (25^th^-75^th^ centile) | 3.67 (0.98-9.88) | 2.74 (0.56-6.86) | 6.52 (3-14.68) | **Kruskal-Wallis *χ^2^=*59.8, df = 1, p<0.0001** | **<0.0001** |
| Adjusted inpatient admission days (inpatient admission days/time with CAMEO in days)*2,500, median (25^th^-75^th^ centile) | 10.37 (0.98-213.21) | 3.62 (0.56-48.46) | 119.05 (5.7-1019.18) | **Kruskal-Wallis *χ^2^=*108.06, df = 1, p<0.0001** | **<0.0001** |
| CRHTT admission rate (N of CRHTT admissions/time with CAMEO in days)*2,500, median (25^th^-75^th^ centile) | 3.48 (0.74-9.19) | 3.31 (0.68-8.13) | 4.16 (1.14-11.14) | **Kruskal-Wallis *χ^2^=*5.02, df = 1, p=0.02** | **0.03** |
| Adjusted days with CRHTT (days with CRHTT/time with CAMEO in days)*2,500, median (25^th^-75^th^ centile) | 5.94 (0.71-38.39) | 4.38 (0.68-35.21) | 9.99 (1.01-45) | **Kruskal-Wallis *χ^2^=*4.6, df = 1, p=** **0.03** | **0.04** |
| *Intensity of care index* (PCA of Intensity Measures Factor 1), median (25^th^-75^th^ centile) | -0.18 (-0.21--0.11) | -0.2 (-0.22--0.14) | -0.15 (-0.2--0.06) | **Kruskal-Wallis *χ^2^=*65.6, df = 1, p<0.0001** | **<0.0001** |

^1^ a Kruskal-Wallis rank sum test was used for skewed continuous data which could not be normalised with log-transformation

###### Supplementary Table 2: Baseline psychiatric symptom severity, overall and by clinical outcome

|  | **Sample with longitudinal psychiatric outcomes**  **N=749** | | **Good outcome (discharged to primary care from EIS)**  **N=**447 (59.68%) | **Poor outcome (continued psychiatric care after EIS)**  **N=**302 (40.32%) |  |
| --- | --- | --- | --- | --- | --- |
| **Characteristic** | **N with data** | **Median (25^th^-75^th^ centile)** | **Median (25th-75th centile)** | **Median (25th-75th centile)** | **Difference between groups - test statistic^1^** |
| Short Warwick-Edinburgh Mental Wellbeing Scale (SWEMWBS) | 155 | 20 (16.5-25) | 21 (17-25) | 18 (15-24) | K-W *χ^2^*= 2.63, df = 1, p=0.10 |
| Clinical Global Impression (CGI) | 116 | 5 (4-5.25) | 5 (4-5.5) | 5 (4-5) | K-W *χ^2^*= 0.26, df = 1, p=0.61 |
| Brief psychiatric rating scale (BPRS) | 115 | 58 (47-66) | 56 (44.5-64.5) | 61 (50.75-67) | K-W *χ^2^*= 2.78, df = 1, p=0.10 |
